# Beyond Effectiveness: Impact of Cladribine on Patient-Reported Outcomes and Quality of Life in Latin American Populations With Multiple Sclerosis

**DOI:** 10.7759/cureus.99854

**Published:** 2025-12-22

**Authors:** Bernardita Soler, Claudia Cárcamo, Juan De La Barra, Irene Treviño-Frenk, Adriana Carra, Alejandra Martinez, Patricio Correa, Adolfo Del Canto, Carolina Pelayo, Lorena Garcia, Ana Reyes, Macarena Vasquez, Ethel Ciampi

**Affiliations:** 1 Neurology, Pontificia Universidad Católica de Chile, Santiago, CHL; 2 Neurology, Hospital Sotero del Rio, Santiago, CHL; 3 Neurology, Instituto Nacional de Ciencias Médicas y Nutrición "Salvador Zubrian" (INCMNSZ), Mexico, MEX; 4 Neurology, Hospital Británico, Fundación Favaloro, Buenos Aires, ARG; 5 Neurology, Hospital Nacional Profesor Alejandro Posadas, El Palomar, ARG; 6 Neurology, Hospital de Especialidades Carlos Andrade Marín, Quito, ECU; 7 Neuroimmunology, Pontificia Universidad Católica de Chile, Santiago, CHL; 8 Neurology, Red de Salud UC-Christus, Santiago, CHL

**Keywords:** cladribine, latin america, patient-reported outcomes, real-world evidence, safety

## Abstract

Background: This multicenter, prospective, observational study evaluated the real-world effectiveness, safety, and impact on patient-reported outcomes (PROs) and functionality of cladribine (CLAD) in 167 relapsing multiple sclerosis (RMS) patients from Chile, Mexico, Argentina, and Ecuador between 2019 and 2024.

Methods: Patients were assessed for clinical relapses, magnetic resonance imaging (MRI) activity, and disability progression. A subgroup underwent additional cognitive, motor, and PRO evaluations.

Results: The annualized relapse rate (ARR) significantly decreased from 0.65 ± 0.6 pre-treatment to 0.12 ± 0.4 in year one, 0.08 ± 0.3 in year two, 0.06 ± 0.3 in year three, and 0.15 ± 0.5 in year four. No evidence of disease activity-3 (NEDA-3) was achieved by 68% (n = 100/147), 77% (n = 89/115), 86% (n = 61/71), and 82% (n = 37/45) of patients in years one, two, three, and four, respectively. Common adverse events were mild infections and lymphopenia (grade I 33% n = 48, grade II 30% n = 44, grade III 8% n = 12, and grade IV 0.01% n = 1 in year one). No treatment delays occurred due to lymphopenia. Two patients had healthy newborns after becoming pregnant between CLAD courses. Significant improvements were observed in visual memory, verbal memory, and anxiety levels at two years.

Conclusion: CLAD demonstrates an effectiveness and safety profile consistent with clinical trials in Latin American RMS populations, also improving PROs and functional outcomes, supporting its use as a viable treatment option.

## Introduction

Multiple sclerosis (MS), a chronic, inflammatory, immune-mediated disease, causes demyelination and neurodegeneration within the central nervous system (CNS) [1]. It is a leading cause of severe neurological disability in young adults and one of the most common neurological disorders worldwide, significantly impacting physical function, cognition, health-related quality of life (HRQoL), and employment [2-4]. Relapsing MS (RMS), marked by periodic acute exacerbations (relapses) followed by periods of clinical stability, affects approximately 85% of MS patients. These relapses may result in partial or complete recovery of neurological deficits.

Various therapies are available for RMS, and the World Health Organization (WHO) has included cladribine (CLAD) as the only oral therapy for MS in its List of Essential Medicines [5]. Oral CLAD was approved with different indications in Latin America. Some countries allow starting naive patients with highly active disease, while others can only access CLAD as a second-line treatment after treatment failure or severe adverse events.

While randomized controlled trials are essential for new drug approvals due to their controlled populations and ideal conditions, real-world MS cohorts can provide valuable insights that these trials may not capture. Therefore, real-world data can answer questions that randomized trials do not address.

We aimed to conduct a longitudinal observational study across Latin America (Chile, Argentina, Mexico, and Ecuador) to analyze real-world evidence of CLAD treatment, focusing on its effectiveness and safety. In Chile specifically, the protocol incorporated patient-reported outcomes (PROs) and motor and cognitive assessments to evaluate the impact of CLAD on patient quality of life and functionality.

## Materials and methods

This is a multicenter, prospective, observational study that included consecutive patients with RMS undergoing clinical care at the UC-Christus Clinical Hospital or Dr. Sótero del Río Hospital, in Chile; Hospital de Especialidades Carlos Andrade Marín, in Ecuador; Instituto Nacional de Ciencias Médicas y Nutrición "Salvador Zubrian" (INCMNSZ), México; and Hospital Británico, Fundación Favaloro, Hospital Nacional Alejandro Posadas, El Palomar, Argentina.

The inclusion criteria required patients to receive at least one dose of CLAD between 2019 and 2024. Due to Chilean regulations, only patients who had initially received platform treatment and had failed or had experienced a serious adverse effect were allowed [6]. Excluding Chile, all countries received regulatory approval to use CLAD for naive patients. Patients had to be regularly monitored at one of the centers and be invited to participate. The only exclusion criterion was the desire not to participate in the clinical study, which did not affect the care or pharmacovigilance of patients who chose not to participate.

The clinical data included age, time since disease onset, previous disease-modifying therapies (DMTs), and after starting CLAD, Expanded Disability Status Scale (EDSS) scores [7], clinical relapses, and magnetic resonance imaging (MRI) at six months, 12 months, and 24 months. Disability progression was determined by an increase of 1.5 points if the initial EDSS = 0, (b) 1 point if the initial EDSS was between >0 and <6, or (c) 0.5 points if the initial EDSS ≥ 5.5, confirmed for at least six months in the absence of a relapse and maintained throughout the follow-up [8-10]. The appearance of new T2 lesions or new gadolinium-enhanced T1 lesions defined MRI activity [11]. No evidence of disease activity (NEDA) was defined as the absence of disability progression, absence of clinical relapses, and absence of MRI activity (NEDA-3) [8,12,13]. The annualized relapse rate (ARR) was calculated by dividing the total number of relapses by the total time of each patient at risk for relapse during the 12 months of evaluation or early termination in patients who withdrew from the study [8,13,14].

All patients recruited in Chile underwent a comprehensive functional evaluation before the start of CLAD, in the first year of treatment, and upon completing the second year of treatment with EDSS, Symbol Digit Synthesis Test (SDMT), California Verbal Learning Test (CVLT), Brief Visuospatial Memory Test-Revised (BVMT-R), Three-Second Paced Auditory Consecutive Addition Test (PASAT-3), Nine-Hole Peg Test (9HPT), Timed 25-Foot Walk Test (T25FWT), Multiple Sclerosis Impact Scale-29 (MSIS29), Fatigue Severity Scale (FSS), Beck Depression Inventory (BDI), and Beck Anxiety Inventory (BAI) [7,15-27].

Statistical evaluation was performed using SPSS v21 (IBM Corp., Armonk, NY, US); for ordinal variables, median and range were obtained, and for numerical variables, mean and standard deviation were determined. In the study comparing medians, non-parametric tests were used with related variables (Wilcoxon test). Results were considered significant with a p < 0.05. Effect sizes were calculated with Hedges’ g. Patients signed a written informed consent approved by the local Ethics Committee.

## Results

Baseline characteristics

A total of 167 patients were included in the study. Seventy-two patients were from Chile, 69 from Mexico, 16 from Argentina, and 10 from Ecuador. The cohort comprised 74% women (n = 123/167), with a mean age at onset of disease of 28 years (range: 12-62 years) and a mean age at onset of CLAD of 34.5 years (range: 18-71 years). The mean disease duration was 7 ± 7 years (range: 0-47 years). The median EDSS score at the start of treatment was 2 (range: 1-6).

Regarding disease phenotypes, one patient had a diagnosis of clinical isolated syndrome, one had radiologically isolated syndrome, 83 presented with relapsing-remitting MS, 14 had active secondary progressive MS, and one had inactive secondary progressive MS. The ARR before the initiation of CLAD was 0.65 ± 0.6. The mean number of prior treatments before starting CLAD was one (range: 0-4). Among the patients, 27% (n = 45/167) were treatment-naive. Reasons for initiating CLAD among the remaining patients included treatment failure (40%, n = 67/167), adverse effects (11%, n = 19/167), and other factors (22%, n = 36/167) such as treatment de-escalation, desire for medium-term pregnancy, or prolonged travel. Among the total cohort, 45 patients (27%) were transitioning from interferon or glatiramer acetate, 21 (13%) from teriflunomide or dimethyl fumarate, 25 (15%) from fingolimod, two (1%) from natalizumab, two from alemtuzumab (1%), one from cyclophosphamide (1%), and 25 (15%) from anti-CD20 therapies (Table 1).

**Table 1:** Baseline demographic and clinical characteristics of included patients W: women; M: male; CLAD: cladribine; SD: standard deviation; RIS: radiologically isolated syndrome; CIS: clinically isolated syndrome; RR: relapsing-remitting; SPa: active secondary progressive; SP: secondary progressive; EDSS: Expanded Disability Status Scale; ARR: annualized relapse rate; Gd+: gadolinium-enhancing lesions; DMT: disease-modifying therapy; IFN/GA: interferon/glatiramer acetate; TER/DMF: teriflunomide/dimethyl fumarate; FTY: fingolimod; NTZ: natalizumab; RTX/OCR: rituximab/ocrelizumab; ALEM: alemtuzumab; CYC: cyclophosphamide

Variable	N = 167
Sex n(%) W:M	123 (74):44 (26)
Age at onset median (range) (years)	28 (12-62)
Age at CLAD start median (range) (years)	34.5 (18-71)
Disease duration at CLAD start mean ± SD (range) (years)	7 + 7 (0-47)
Phenotype RIS/CIS/RR/SPa/SP (%)	1/1/83/14/1
Baseline EDSS median (range)	2 (0-8)
Baseline ARR mean ± SD	0.65 + 0.6
Baseline Gd+ n(%)	99 (59)
Baseline new T2 n(%)	99 (59)
Number of previous treatments median (range)	1 (0-4)
Reasons for CLAD initiation n(%)	
Naive/highly active disease	45 (27)
Treatment failure	67 (40)
Adverse events with previous DMT	19 (11)
Other (e.g., pregnancy and travel)	36 (22)
Previous DMT n(%)	
Naive	45 (27)
IFN/GA	45 (27)
TER/DMF	21 (13)
FTY	25 (15)
NTZ	2 (1)
RTX/OCR	25 (15)
ALEM	2 (1)
CYC	1 (1)

Effectiveness outcomes

All 167 patients received the first dose of CLAD. During the first year, eight patients (4.8%) required a treatment switch due to multifocal relapses; no cases of breakthrough disease were observed during the second year of follow-up. Among the 147 patients who completed the first year of treatment, the mean ARR was 0.12 ± 0.4. Fifteen patients (10%) exhibited disability progression during the first year, 20 patients (14%) showed MRI activity, and 12 patients (8%) experienced a clinical relapse. A total of 100 patients (68%) achieved NEDA-3 by the end of the first year (Table 2).

**Table 2:** Effectiveness of cladribine ARR: annualized relapse rate; MRI: magnetic resonance imaging; EDSS: Expanded Disability Status Scale; NEDA: no evidence of disease activity; n: number; SD: standard deviation

Completed 1st year follow-up	n = 147	Completed 2nd year follow-up	n = 115
ARR mean ± SD	0.12 + 0.4	ARR mean ± SD	0.08 + 0.3
Relapses n(%)	12 (8)	Relapses n(%)	6 (5)
MRI activity n(%)	20 (14)	MRI activity n(%)	11 (10)
EDSS progression n(%)	15 (10)	EDSS progression n(%)	9 (8)
NEDA-3	68%	NEDA-3	56%
Completed 3rd year follow-up	n = 71	Completed 4th year follow-up	n = 45
ARR mean ± SD	0.06 + 0.3	ARR mean ± SD	0.15 + 0.5
Relapses n(%)	2 (3)	Relapses n(%)	3 (7)
MRI activity n(%)	6 (8)	MRI activity n(%)	3 (7)
EDSS progression n(%)	2 (3)	EDSS progression n(%)	2 (4)
NEDA-3	86%	NEDA-3	70%

One hundred fifteen patients completed the second year of CLAD with an ARR of 0.08 ± 0.3. Nine patients (8%) experienced disability progression during the second year, and 11 patients (10%) exhibited MRI activity. In this cohort, 89 patients (77%) reached NEDA-3 by the second year (Table 2). Seventy-one patients completed the third year of treatment with an ARR of 0.06 ± 0.3. During this period, two patients (3%) showed disability progression, six patients (8%) exhibited MRI activity, and two patients (3%) experienced clinical relapses. Sixty-one patients (86%) achieved NEDA-3 by the third year (Table 2). Forty-five patients completed the fourth year of follow-up with an ARR of 0.15 ± 0.5. Two patients (4%) experienced disability progression, three patients (7%) exhibited MRI activity, and three patients experienced clinical relapses. Thirty-seven patients (82%) reached NEDA-3 by the fourth year (Table 2). During the third and fourth years of treatment, a total of nine patients changed therapy due to treatment failure, all switching to anti-CD20 therapies.

Safety

No adverse events related to oral medication tolerance were reported among the patients. Laboratory evaluations revealed grade I lymphopenia in 48 patients (33%), grade II in 44 patients (30%), grade III in 12 patients (8%), and grade IV in one patient (0.01%) during the first year. No patients experienced treatment delays due to persistent lymphopenia. In the second year, grade I lymphopenia was observed in 18 patients (16%), grade II in 31 patients (27%), and grade III in six patients (5%) (Table 3). Patients who presented with lymphopenia below 500 cells/mm^3^ received prophylaxis for *Pneumocystis jirovecii* and prophylaxis against herpes according to local practices. Patients with a history of herpes zoster or recurrent herpes received prophylaxis with absolute lymphocyte count (ALC) at less than 700 cells/mm^3^.

**Table 3:** Lymphopenia observed after the first and second courses of cladribine

Lymphopenia (cells/mm^3^)/months	1	2	3	6	9	12	13	14	15	18	21	24
Grade I (800-900)	5	10	7	13	6	7	4	0	6	6	0	2
Grade II (500-799)	12	17	10	4	0	1	15	0	8	6	0	2
Grade III (200-499)	4	3	2	3	0	0	2	0	3	1	0	0
Grade IV (<200)	1	0	0	0	0	0	0	0	0	0	0	0

The most common adverse events were infections (COVID-19, herpes zoster, urinary tract infections, and vaginitis), all mild with complete recovery. One male patient aged 53 experienced a myocardial infarction (the patient had a known cardiovascular comorbidity). Two patients died, one from an intracranial hemorrhage and another from vasculitis; neither of them was attributed to CLAD use. Secondary autoimmune conditions were observed in one (0.01%) male patient during the second year of follow-up (autoimmune thyroid disorder). No cases of progressive multifocal leukoencephalopathy (PML) were reported. One patient developed endometrial cancer during the first year, one patient had papillary thyroid cancer in the second year, and one patient was diagnosed with melanoma during the second year (Table 4). We highlight that all of these patients were diagnosed within screening programs suggested by their treating neurologists. Finally, two patients became pregnant between the first and second doses of CLAD, both resulting in healthy newborns (Table 4).

**Table 4:** Adverse events during follow-up *Autoimmune thyroiditis **One central nervous system vasculitis, one hemorrhagic stroke

Infections (n)	First year	Second year	Total
COVID-19 infection	2	4	6
Upper respiratory tract infections	4	0	4
Bacterial vaginitis	1	0	1
Urinary tract infection	1	1	2
Herpes zoster	2	6	8
Secondary autoimmune disorders*	0	1	1
Acute myocardial infarction	0	1	1
Pregnancy	2	0	2
Seizures	1	0	1
Endometrial cancer	1	0	1
Papillary thyroid cancer	0	1	1
Melanoma	0	1	1
Death**	2	0	2

Functional evaluation and PROs

Statistically significant improvements were observed in visual episodic memory (BVMT-R: median baseline 26 (8-36) vs. 30 (16-36), p = 0.049, Hedges’ g 0.7), verbal episodic memory (CVLT-VIII: median baseline 64 (26-77) vs. 69 (56-76), p = 0.05), and anxiety levels (BAI: median baseline 6 (1-16) vs. 3 (0-12), p = 0.03, Hedges’ g 0.548). This improvement in visual memory was further reflected by an increase from the 75th percentile at baseline to the 95th percentile at the end of follow-up.

Trends toward improvement were also noted in walking speed (T25FWT: median baseline 6.2 seconds (5-13) vs. six seconds (4-8), p = 0.053, Hedges’ g 0.518) and overall disease impact (MSIS29 total: median baseline 49.5 (29-92) vs. 36 (29-69), p = 0.059; Hedges’ g 0.644, driven by an improvement of the physical impact). Other evaluated parameters, including EDSS, SDMT, PASAT, 9HPT (dominant and non-dominant upper limbs), MSIS29 psychological subscale, FSS, and BDI, remained stable without significant changes or progression of disability at two years (Table 5).

**Table 5:** Longitudinal functional valuation of the Chilean cohort **p < 0.05 indicates statistical significance *Trends were observed toward improvement in CVLT, T25FWT, and MSIS29 total and phys EDSS: Expanded Disability Status Scale; SDMT: Symbol Digit Modality Test; CVLT: California Verbal Learning Test; BVMT-R: Brief Visual Memory Test-Revised; PASAT: Paced Auditory Serial Addition Test; 9HPT-dom: Nine-Hole Peg Test (dominant upper limb); 9HPT-non dom: 9-Hole Peg Test (non-dominant upper limb); T25FWT: Timed 25-Foot Walk Test; MSIS29: Multiple Sclerosis Impact Scale-29; MSIS29 phys: Multiple Sclerosis Impact Scale-29 (physical subscale); MSIS29 psyc: Multiple Sclerosis Impact Scale-29 (psychological subscale); FSS: Fatigue Severity Scale; BDI: Beck Depression Inventory; BAI: Beck Anxiety Inventory. Hedges’ g: an effect size of 0.2 to 0.3 is considered a “small” effect, 0.5 a “medium effect,” and ≥0.8 a “large effect”

Outcome	Baseline median (range)	2-year follow-up median (range)	p	Hedges’ g
EDSS	2.0 (0-3)	2.0 (0-6)	0.799	0.069
SDMT	57 (32-78)	61 (33-86)	0.209	0.342
CVLT	64 (26-77)	69 (56-76)	0.050*	0.403
BVMT-R	26 (8-36)	30 (16-36)	0.049**	0.700
PASAT	48 (12-58)	51 (34-59)	0.331	0.019
9HPT-dom seconds	19 (16-40)	19 (15-38)	0.790	0.145
9HPT-non dom seconds	20 (17-26)	20 (16-31)	0.930	0.283
T25FWT seconds	6.2 (5-13)	6 (4-8)	0.053*	0.518
MSIS29 total	49.5 (29-92)	36 (29-69)	0.059*	0.644
MSIS29 phys	27.5 (20-61)	22.5 (20-56)	0.052*	0.555
MSIS29 psyc	18 (9-31)	13.5 (9-33)	0.190	0.657
FSS	15 (9-54)	20 (9-57)	0.799	0.154
BDI	8 (0-20)	10 (0-21)	0.201	0.399
BAI	6 (1-16)	3 (0-12)	0.04**	0.548

## Discussion

This multicenter, longitudinal observational study offers valuable real-world evidence on CLAD use within a Latin American cohort. Its significant strengths include the four-year follow-up, the inclusion of PROs, and functional assessments, all of which are particularly relevant given regional variations in MS management and treatment access.

Real-world evidence is critical for understanding a treatment's use, safety, and efficacy beyond the controlled setting of clinical trials. The study's findings on effectiveness are consistent with existing data. The observed treatment discontinuation rate of 4.8% aligns with a 2023 systematic review by Oreja-Guevara et al., which reported rates from 0% to 11% [28]. That same review noted a low incidence of disease reactivation (1.1%-21.9%), particularly in patients switching from antilymphocyte trafficking agents [28]. This was reflected in our study, where all patients who experienced breakthrough activity were switching from fingolimod.

The results also align with major clinical trials like CLARITY and CLARITY Extension, which reported significant reductions in relapse rates and disability progression [8,13,29]. In our cohort, the ARR decreased significantly in the first year, with 68% of patients achieving NEDA-3. This disease control was sustained, with a high percentage of patients maintaining NEDA-3 status over the four-year follow-up. The achievement of NEDA-3 is considered a critical measure of comprehensive disease control.

The safety profile in this cohort was consistent with that observed in clinical trials [13,30,31]. Lymphopenia, an expected side effect of CLAD's mechanism of action, was the most frequently reported adverse event, occurring in 33% of patients in the first year. A small proportion experienced grade III and IV lymphopenia. Importantly, no treatment delays or dose reductions were required due to lymphopenia, and the overall adverse event profile was mild, consisting mainly of infections for which patients received prophylaxis per clinical guidelines.

Early cancer diagnosis in our population is a noteworthy point, highlighting the standard screening carried out in different countries and by professionals prescribing DMTs. Thus, all patients are recommended an annual dermatology check-up, women over 40 years old should have a mammogram (which can start earlier if there is a family history of cancer), cervical cancer screening is recommended for women between 25 and 64 years old, and colorectal cancer screening with colonoscopy is performed according to risk factors and age. Additionally, it is suggested to evaluate other risk factors for cancer, which include patients over 50 years old, with a family history of neoplasms, with more than 10 years of treatment with DMT or who have had at least two therapy changes, or who have received therapy with a confirmed risk of cancer [32]. Data from long-term follow-up registries of clinical trial patients confirmed that the incidence of cancer did not increase with the duration of therapy and was not higher than with other DMTs [13,31,32]. Two deaths occurred in the study, but they were not considered directly attributable to the drug. Reassuringly, no cases of progressive multifocal leukoencephalopathy were reported. Additionally, one patient developed an autoimmune thyroid disorder during the second year of follow-up, which is in line with reports of secondary autoimmune diseases in other studies involving immunomodulatory therapies [8]. These outcomes highlight the need for continued vigilance and appropriate management of comorbidities in MS patients undergoing immunosuppressive treatment.

The study also highlights the relevance of evaluating CLAD's effectiveness and safety in diverse populations, particularly in Latin American countries, where MS management may vary significantly due to differences in access to healthcare, healthcare systems, and treatment availability. In our cohort, a substantial proportion of patients had previously been treated with other DMTs, including interferons, glatiramer acetate, and anti-CD20 therapies, underscoring the real-world setting where many patients are treated after experiencing treatment failure or adverse events with first-line therapies. Notably, 27% of patients were treatment-naive at the time of starting CLAD, which may reflect the expanding access to newer therapies in the region.

In Latin America, the availability of DMTs like CLAD is often restricted by country-specific regulatory approvals, and patient access can vary greatly. In some countries, CLAD is available as a first-line treatment for highly active RMS, while in others, it is reserved for patients who have failed other treatments. This disparity in treatment access presents challenges for clinicians who must navigate complex treatment algorithms and consider local healthcare resources when making therapeutic decisions.

A unique contribution of this research is its exploration of CLAD's effect on outcomes beyond NEDA-3, such as cognitive function, anxiety, and quality of life. Using a range of functional and patient-reported assessments, the study found significant improvements in visuospatial memory (BVMT-R), verbal episodic memory (CVLT-VIII), and anxiety levels (BAI). A positive trend was observed in walking speed (T25FWT) and overall quality of life (MSIS29), mainly driven by the physical component. Other parameters remained stable, indicating no progression of disability, which is a positive outcome. These findings align with those reported by Conway et al. in 2025, with no significant differences in manual dexterity or cognitive speed processing tests. Additionally, their study showed significant improvements in gait speed and NeuroQoL fatigue scores, favoring the CLAD-treated group [33]. These improvements are especially noteworthy as they were observed while patients were receiving treatment during the COVID-19 pandemic, a period known to have negatively impacted the mental health and quality of life of MS patients [34].

The observed improvement in cognitive measures is likely underpinned by the effective control of inflammatory activity-specifically, fewer relapses and new MRI lesions-seen during follow-up. This control may have allowed patients to compensate for or recover from acute damage that occurred around the time of CLAD initiation, which is supported by the high incidence of new T2/Gd+ lesions on baseline MRI.

Conversely, the improvement in anxiety metrics could be attributed to CLAD's convenient, home-based administration, which involves only two five-day treatment periods per year, for two years, coupled with its favorable safety profile and low requirement for pharmacovigilance. This finding aligns with clinical trial results reported by Brochet et al. and Afolabi et al., which demonstrated significant improvements in mean MSQoL-54 physical and mental health composite scores, as well as the EQ-5D index [35,36].

In 2023, the WHO's Model Essential Medicines List incorporated only three medications for treating MS, with CLAD being the sole oral agent. Noted for its favorable benefit-to-risk profile, CLAD's inclusion, alongside the other medicines, on this global policy tool aims to enhance worldwide access to MS treatment. The Essential Medicines List serves as a guide for governments, health facilities, and procurers in selecting cost-effective medicines [5].

Our study, despite promising results, has several limitations. As an observational study, it inherently lacks the control and randomization of a clinical trial, making its results susceptible to biases like selection bias and confounding factors. Additionally, the cohort's heterogeneity in disease phenotype and prior treatment history may have influenced treatment outcomes, and the small sample size prevents a more detailed subgroup analysis (e.g., comparing naive patients versus those with treatment failure, or analysis by country). Future research should include head-to-head trials comparing CLAD to other therapies and studies examining long-term outcomes in larger, more diverse populations to improve our understanding of its role in treating RMS and improving functional outcomes beyond NEDA-3.

## Conclusions

This multicenter, longitudinal observational study provides real-world evidence supporting the effectiveness and safety of CLAD for treating MS in Latin American populations. The study demonstrates CLAD's potential for sustained disease control, evidenced by a significant reduction in relapse rates and the achievement of NEDA-3 over four years. The observed safety profile is consistent with clinical trial data, showing manageable adverse events and no treatment delays caused by lymphopenia. Furthermore, the study uniquely highlights improvements in PROs, including cognitive function, anxiety levels, and walking speed, alongside overall quality of life. These findings underscore CLAD's viability as an MS treatment option, especially in varied real-world settings with diverse patient access and healthcare systems. Future research should investigate long-term outcomes and compare CLAD with other therapies to further validate these results and optimize treatment strategies for RMS patients.
